# RC Structures Strengthened by an Iron-Based Shape Memory Alloy Embedded in a Shotcrete Layer—Nonlinear Finite Element Modeling

**DOI:** 10.3390/ma13235504

**Published:** 2020-12-03

**Authors:** Neda Dolatabadi, Moslem Shahverdi, Mehdi Ghassemieh, Masoud Motavalli

**Affiliations:** 1 School of Civil Engineering, University of Tehran, Tehran 1417466191, Iran; n.dolatabadi@ut.ac.ir (N.D.); m.ghassemieh@ut.ac.ir (M.G.); Masoud.Motavalli@empa.ch (M.M.); 2 Swiss Federal Laboratories for Materials Science and Technology, Empa, 8600 Dubendorf, Switzerland

**Keywords:** nonlinear finite element, shape memory alloy, strengthening, pre-stressing, concrete

## Abstract

Shape memory alloys (SMAs) have been widely used in civil engineering applications including active and passive control of structures, sensors and actuators and strengthening of reinforced concrete (RC) structures owing to unique features such as the shape memory effect and pseudo-elasticity. Iron-based shape memory alloys (Fe-SMAs) have become popular in recent years. Use of iron-based SMAs for strengthening RC structures has received attention in the recent decade due to the advantages it presents, that is, no ducts or anchor heads are required, friction losses do not occur and no space is needed for a hydraulic device to exert force. Accordingly, Fe-SMAs embedded in a shotcrete layer have been used for pre-stressing RC beams at Empa. The aim of this study is to present an approach to model and analyze the behavior of RC members strengthened and pre-stressed with Fe-SMA rebars embedded in a shotcrete layer. The lack of research on developing finite element models for studying the behavior of concrete structures strengthened by iron-based shape memory alloys is addressed. Three-dimensional finite element models were developed in the commercial finite element code ABAQUS, using the concrete damaged plasticity model to predict the studied beams’ load–displacement response. The results of the finite element analyses show a considerably good agreement with the experimental data in terms of the beams’ cracking load and ultimate load capacity. The effects of different strengthening parameters, including SMA rebar diameter, steel rebar diameter and pre-stressing force level on the beam behavior, were investigated based on the verified finite element models. The results were compared. The load-displacement response of an 18-m concrete girder strengthened and pre-stressed with iron-based SMA bars was examined by the developed finite element model as a case study.

## 1. Introduction

Shape memory alloys are a class of materials that have gained popularity in recent decades owing to unique features such as the shape memory effect (SME) and pseudo-elasticity. Numerous studies have addressed the use of these alloys in engineering applications. In civil engineering applications, Ni-Ti based alloys have been widely used in the confinement of columns, dampers, self-centering bridge components and pre-stressing of structural members [1]. 

Iron-based shape memory alloys (Fe-SMAs) are an appropriate alternative for Ni-Ti based alloys in structural applications based on their lower cost, wider thermal hysteresis, good machinability, higher stiffness and the ability to develop high recovery stresses without thermomechanical training. It is noteworthy that the lower cost of Fe-SMAs stems from the lower cost of Fe and the less expensive production cost that can be conducted under atmospheric conditions [2,3,4,5,6,7,8,9,10]. Fe-SMAs have varied compositions; each is different in terms of phase transformation and thermal hysteresis characteristics. Among these, the Fe-Mn-Si alloy has nearly the same tensile strength and coefficient of thermal expansion as stainless steel. Thus, this alloy is suitable for structural applications requiring a material that exhibits the SME [11]. The one-way SME of the Fe-Mn-Si alloy makes it a reasonable option for permanent structural pre-stressing purposes, as it does not undergo phase transformation due to temperature fluctuations after installation. The application of Fe-SMAs in joints is presented in reference [12]. In contrast, thermo-elastic alloys such as Ni-Ti are functional materials that display phase transformation behavior several times. 

Shahverdi et al. experimentally investigated the application of a novel Fe-SMA developed at Empa for near-surface mounted (NSM) strengthening; their studies [13] showed that such strengthening techniques were successful. In another study [14], they demonstrated the strengthening and stiffening potential of reinforced concrete beams with ribbed Fe-SMA bars embedded in an applied shotcrete layer.

SMA-reinforced structures have been simulated through finite element models in few studies [5,15,16]. Abouali et al. [5] studied the effect of strengthening reinforced concrete beams with near-surface mounted (NSM) Fe-SMA strips using 3-D nonlinear finite element model in ABAQUS. Comparison of the results with experimental data showed significant accuracy of the model. Ghassemieh et al. [15] investigated the effect of pseudo-elastic Ni-Ti reinforcement on the behavior of concrete shear walls by performing time history analysis on the finite element models of the structures. The results demonstrated that Ni-Ti reinforcement reduced the residual deformations of the shear walls. They also studied the effect of pretension in SMA bars embedded in the shear walls. The results proved that the use of SMA in concrete shear walls increased stiffness compared to walls reinforced with regular steel bars or non-pretensioned SMA bars. Ruiz- Pinilla et al. [15] proposed an analytical stress-strain curve for modeling the Fe-SMA strip behavior and implemented the curve into a nonlinear finite element model of concrete beams strengthened in shear by the same strips. Results of the developed numerical model showed very good accuracy in simulation of beam’s response. Alam et al. [17] developed a finite element model of the joints tested by Youssef and his co-workers [12]. One joint was reinforced with steel rebars, while the other was reinforced with SMA bars in the plastic hinge region of the beam. Alam et al. used the one-dimensional super-elastic model for defining SMA behavior, as proposed by Auricchio et al. [18]. The results from the finite element model including the load displacement, the moment–rotation relationship and the energy dissipation capacity demonstrated reasonable agreement with the experimental data. Abdulridha et al. [19] developed a preliminary model for defining the hysteretic behavior of SMAs. The developed model was implemented in a finite element model of concrete beams reinforced with SMA bars in the FE-code VecTor2. The model was successful in predicting the load displacement curves of the beams under cyclic loading. Malagisi et al. [20] developed a new computational approach to model the behavior of concrete beams equipped with SMA actuators to repair cracks. A uniaxial model was used to simulate the super-elastic and shape memory effects of the SMA bars. They adopted this model along with the non-local damage and plasticity model for micro-cracking of concrete and a transition approach from damage to fracture for macro-cracks, to create finite element models of beams reinforced with SMA bars. The beams were tested under three-point bending conditions. The numerical results demonstrated that the approach successfully captured the behavior of the SMA-reinforced beams.

Although many publications have numerically modeled Ni–Ti behavior in civil engineering structures using the inherent SME and super-elasticity of the Ni–Ti alloy, few researchers [21] have addressed the simulation of Fe-SMAs, which are characterized by their noticeably different properties. To the best of the authors’ knowledge, modeling the behavior of a ribbed Fe-SMA embedded in a shotcrete layer to strengthen an RC structure has not been studied. 

The main aim of this study is to detail the modeling of RC beams strengthened and pre-stressed with ribbed Fe-SMA bars embedded in a shotcrete layer. The Fe-SMA bars, with a diameter of 8 mm, were pre-strained and fixed to the bottom of the concrete beams. A shotcrete layer was applied to embed the bars. The recovery stress in the embedded Fe-SMA bars was activated by resistive heating [14]. The results of the numerical analysis were compared to the experimental data and the effects of variations in the material parameters were investigated. A parametric study on the steel rebar diameter, SMA bar diameter and pre-stressing force was conducted in the developed and verified FE models to better understand the effect of each parameter on the beam response under a four-point bending load.

## 2. Experimental Program

**Figure 1:**
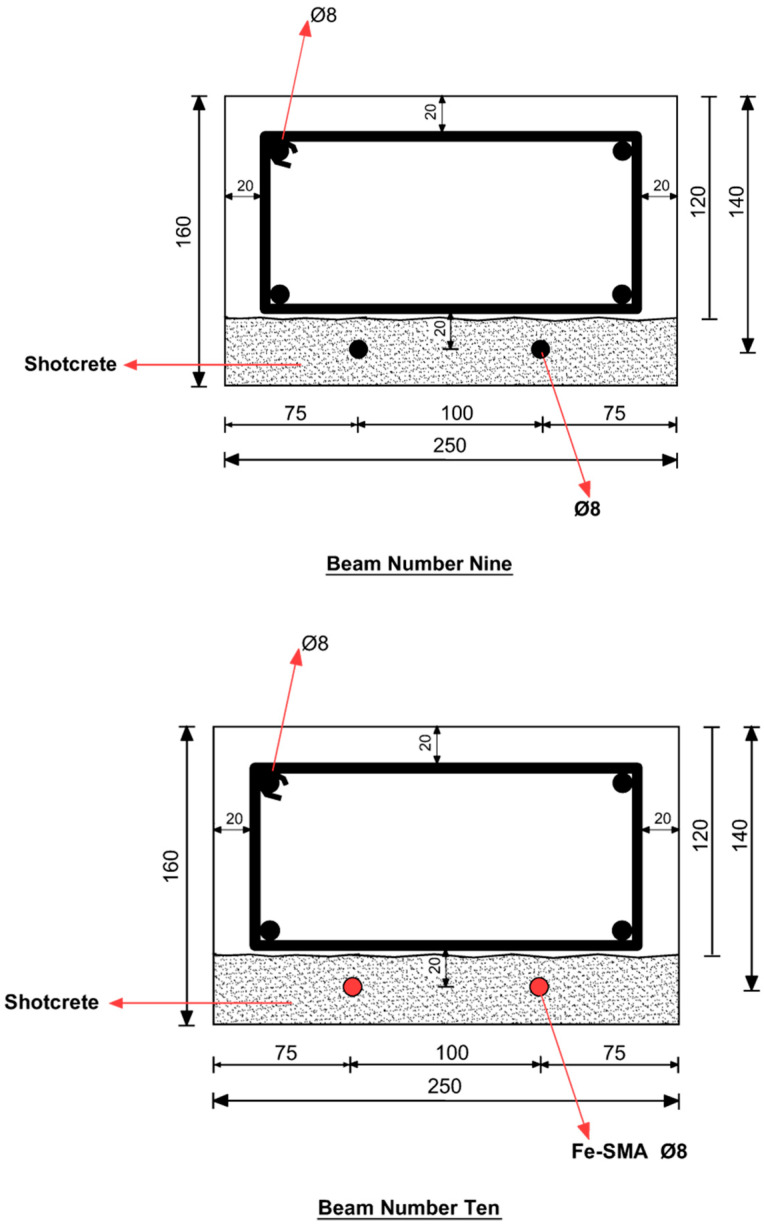

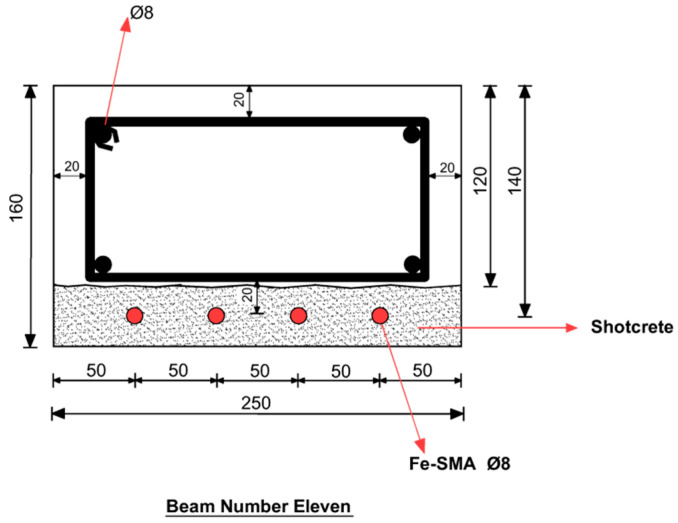
Cross-sections of Beams 9, 10 and 11 (in mm) [14].

**Figure 2:**
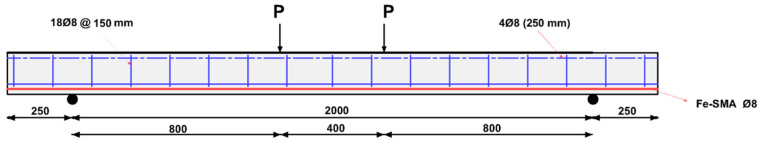
Test set-up of the beams (in mm) [14].

Three concrete beams with a cross-section of 160 mm × 250 mm and a span of 2 m were tested by Shahverdi et al. [14] (Figure 1). Beam 9 was strengthened by two steel rebars; Beams 10 and 11 were strengthened and pre-stressed by two and four ribbed Fe-SMA rebars, respectively. After full curing of the concrete, the lower faces of the beams were roughened to increase friction. The Fe-SMA bars were pre-strained and attached to the roughened surfaces. A 40-mm shotcrete layer was applied on the face embedding the SMA rebars. A recovery stress of approximately 300 MPa was activated by resistive heating, with a temperature of 160 °C applied to the Fe-SMA rebars. Four-point bending loading experiments were conducted to evaluate the effect of strengthening and pre-stressing on the beam behavior, as shown in Figure 2.

## 3. Finite Element Simulation

### 3.1. Material Model for Concrete in ABAQUS

To model the concrete behavior in ABAQUS, three methods are available: the smeared crack model, the brittle cracking model and the concrete damaged plasticity model. In the first method, as the name implies, cracks are not modeled individually on a macroscopic scale. The yield criteria are checked at each integration point and the corresponding stress and strain are calculated accordingly. However, defining damage parameters is not possible using this approach. The brittle cracking model is used when tensile cracking is the dominant behavior in members; compressive crushing is not considered. This model is capable of defining proper concrete behavior in the post-cracking stage but it simply assumes that concrete behaves elastically under compression. Concrete damaged plasticity is a combined model that accounts for both tensile cracking and compressive crushing of concrete. It is designed to predict the nonlinear behavior of concrete using the concepts of damage and plasticity, which are characterized by changes in elastic stiffness and irreversible deformations upon loading [22,23]. 

Numerous models have been proposed to define concrete behavior under uniaxial compression. The model suggested by [24], represented in Equation (1), was used in the current study. The parameter β depends on the shape of the stress–strain curve and is calculated using Equation (2).

(1) $$f_{c}=f_{c}^{\prime }\left[\frac{\beta (\frac{\varepsilon }{\varepsilon_{0}})}{\beta -1+{\left(\frac{\varepsilon }{\varepsilon_{0}}\right)}^{\beta }}\right]$$

where β is determined using Equation (2) and $\varepsilon_{0}$, strain at ultimate strength, is calculated using Equation (3).

(2) $$\beta =\frac{1}{1-\frac{f_{c}^{\prime }}{\varepsilon_{0}E_{it}}}$$

(3) $$\varepsilon_{0}=0.00078f_{c}^{0.25}$$

To determine the direct tensile strength in this simulation, Equation (4) proposed in ACI-318 [25] is used to calculate the direct tensile strength based on the compressive strength.

(4) $$f_{t}^{\prime }=0.33\sqrt{f_{c}^{\prime }}$$

Equation (5) is used to convert flexural tensile strength to direct tensile strength [26] for shotcrete.

(5) $$f_{ctm}=f_{ct,ft}\frac{1.5{\left(h_{b}/h_{0}\right)}^{0.7}}{1+1.5{\left(h_{b}/h_{0}\right)}^{0.7}},$$

where $f_{ct,ft}$ is the flexural tensile strength, $h_{b}$ is the depth of the beam in the flexural tensile strength test and $h_{0}$ is set as 100 mm.

To define the tension stiffening in RC members, the contribution of concrete to the rigidity of RC members even after cracking should be considered [27]. There are two ways to define the tension stiffening behavior of concrete in ABAQUS [22]. It can be defined using the stress–strain relationship or the stress–cracking displacement relationship can be used to define the post-cracking tensile behavior of concrete. When there is a low reinforcement ratio in a beam, the results display mesh-sensitivity. Using the concept of fracture energy and defining the stress–cracking displacement relationship, this problem can be solved and the post-cracking tensile behavior of concrete can be effectively defined. Using the brittle fracture concept, Hillerbourg [28] defined fracture energy ($G_{f}$) as the energy required to open the unit area of a crack. This method is used to define tension stiffening in this simulation; the area under the stress–cracking displacement curve is equal to the fracture energy of concrete as shown by Equation (6) [29].

(6) $$G_{f}=\int_{0}^{\delta_{max}}\sigma_{t}d\delta_{t}$$

where $\sigma_{t}$ is the tensile stress, $\delta_{t}$ is the cracking displacement and $\delta_{max}$ is the maximum cracking displacement. 

Equation (7) was proposed in CEB-FIP 90 [26] to determine the fracture energy of concrete as a function of the maximum aggregate size and compressive strength.

(7) $$G_{f}=G_{fo}{\left(f_{cm}/f_{cmo}\right)}^{0.7}$$

where $G_{fo}$ is the base value of fracture energy that depends on the maximum aggregate size, $f_{cm}$ is the concrete compressive strength and $f_{cmo}$ is set to 10 MPa. Consequently, the fracture energy of the concrete was calculated as 90 N/m. Using this concept, a linear stress–cracking displacement was adopted to define the post-cracking tensile behavior of concrete and shotcrete [22]. 

### 3.2. Modeling the Iron-Based Shape Memory Alloy in ABAQUS

**Figure 3:**
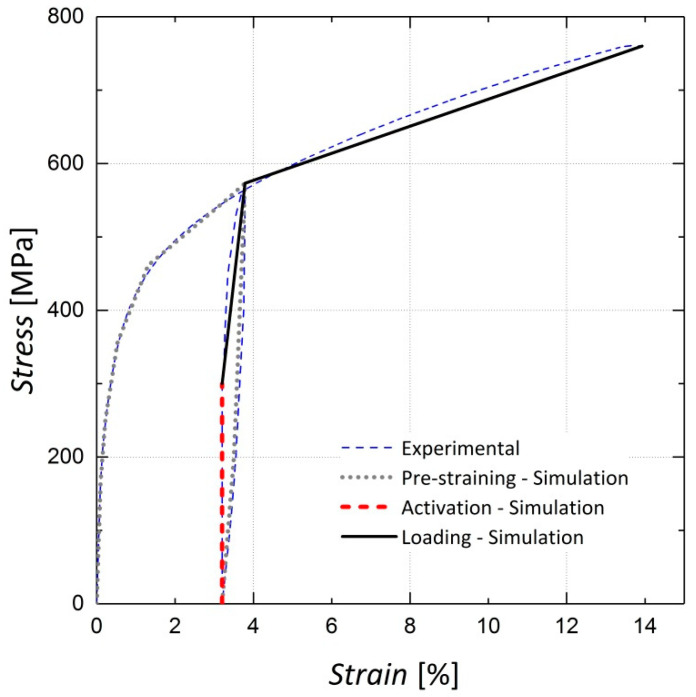
Curve defining the iron-based shape memory alloy (Fe-SMA) tensile behavior in ABAQUS.

The behaviors of Fe-SMA bars with a composition of Fe–17Mn–5Si–10Cr–4Ni–1(V,C) (mass %), were modeled in this study. During pre-straining, the Fe-SMA bars were loaded to a tensile strain of 4% and unloaded to a stress-free state. The bars were anchored to the bottom face of the beam and the shape change was suppressed. The shape memory effect was activated in Fe-SMA bars by heating, resulting in the development of recovery stress due to the enforced deformation constraint. The generated recovery stress was used to pre-stress the concrete members. Figure 3 shows the stress-strain curve from the experiment on Fe-SMA bar, which involves pre-straining, activating and loading compared to the curve defined for simulation of the Fe-SMA bars in ABAQUS. It is noteworthy that unlike many engineering materials, a specific definition for Fe-SMA bars has not yet been implemented in ABAQUS. Thus, a multilinear curve starting from the recovery stress point (the beginning of the black curve in Figure 3) was used to define the activated Fe-SMA behavior embedded in the test beams. As it can be observed in Figure 3, the employed bilinear curve (the black curve) closely resembles the experimental results illustrated by the blue dashed line. A quite similar stress-strain curve for Fe-SMA bar has been cited in [5] where pre-stressed Fe-SMA strips have been used to retrofit concrete beams. 

### 3.3. Finite Element Model Implementation

**Figure 4:**
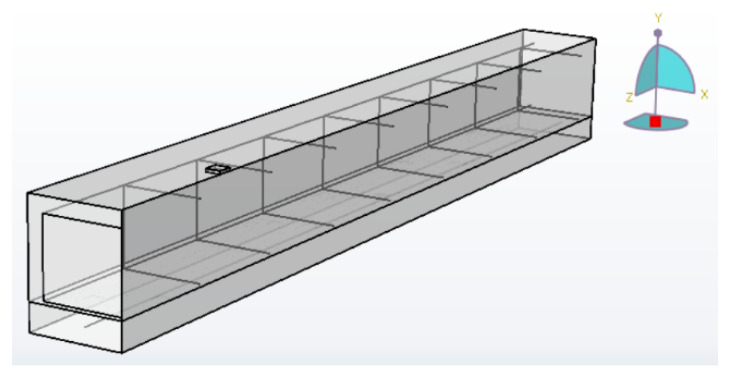
Finite element model of a quarter beam pre-stressed by one Fe-SMA bar in ABAQUS.

ABAQUS/Standard was used in this study as a finite element simulation tool to capture geometric and material nonlinearities. Only a quarter of the beam was modeled, due to symmetry in both the longitudinal and transverse directions, as shown in Figure 4. Eight-node solid elements with reduced integration (C3D8R) were selected for both the concrete and shotcrete parts as these elements prevent the shear-locking phenomenon and yield more accurate results with the proper element size [22]. According to the mesh size sensitivity analyses, 15-mm and 20-mm cubic elements were selected for the reference beam (Beam 9) and beams strengthened with Fe-SMA bars. Due to the small depth of the neutral axis in the tested beams as calculated by cross-section analysis in MATLAB, 10-mm-deep elements were used for the top two layers of mesh to increase the accuracy of the results. Two-node truss elements, T2D2, were used for both the steel and Fe-SMA bars.

According to the experiments, no considerable slippage occurred between the Fe-SMA bars and the surrounding shotcrete or between the steel rebar and the concrete [14]. Thus, the embedded element approach was used to simulate these interactions and a perfect bond was assumed between the Fe-SMA/steel bars and concrete. 

The concrete–shotcrete interaction was a demanding step in this simulation, as the interfacial behavior had to transfer the pre-stressing force to the upper concrete section. To this end, cohesive behavior and rough contact options were used. Without defining a cohesive behavior, the pre-stressing force applied to shotcrete has no effect on the beam response because the concrete and shotcrete lose contact during loading. Rough frictional behavior was used, which assigns an infinite coefficient of friction to the concrete–shotcrete interface. As stated in the ABAQUS manual [22], combining these two approaches prevents penetration of surfaces and intensive slip between them. In addition, the hard contact definition was used to define normal behavior between the two surfaces.

**Figure 5:**
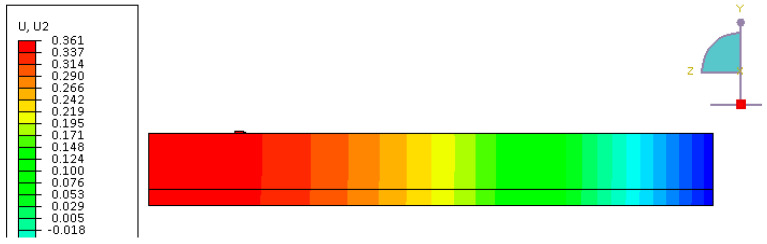
FE results for vertical displacement at the end of the pre-stressing step in ABAQUS.

The pre-stressing force was applied in the initial step before loading using the predefined field option in ABAQUS. The calculation for the upward deflection of the beam pre-stressed with two Fe-SMA bars activated with a recovery stress of 300 MPa is shown in Equation (8). The calculated deflection of 0.36 mm shows a good correlation with the FE result in Figure 5. It should be noted that the results of the experiment show an upward deflection of 0.17 mm during activation. The difference between calculated and observed uplift might stem from the fact that heating the bars before activation of recovery stress results in downwards deflection of the beam. The load was applied to a small steel plate to reduce the stress concentration in the concrete elements located in the vicinity of the load [27].

(8) $$\delta_{pre-stress}=\frac{PeL^{2}}{8EI}=\frac{30159.3\times (140–75.7)\times 2000^{2}}{8\times 34800\times 76357364}=0.36\;\mathrm{mm}$$

## 4. Results and Discussion

### 4.1. Four-Point Bending Test on RC Beams Strengthened by Ribbed Fe-SMA Bars

#### 4.1.1. Concrete Damaged Plasticity (CDP) Parameters 

**Figure 6:**
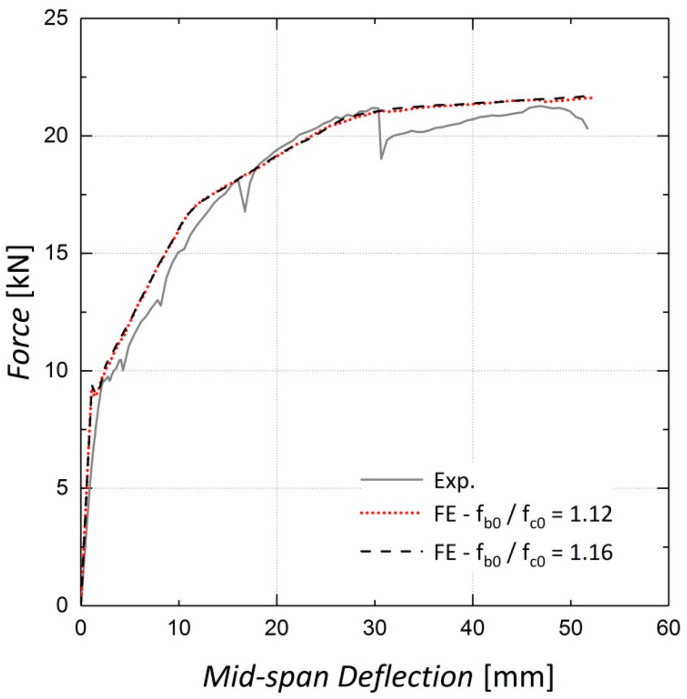
Sensitivity of results to f_b0_/f_c0_—Beam 10.

**Figure 7:**
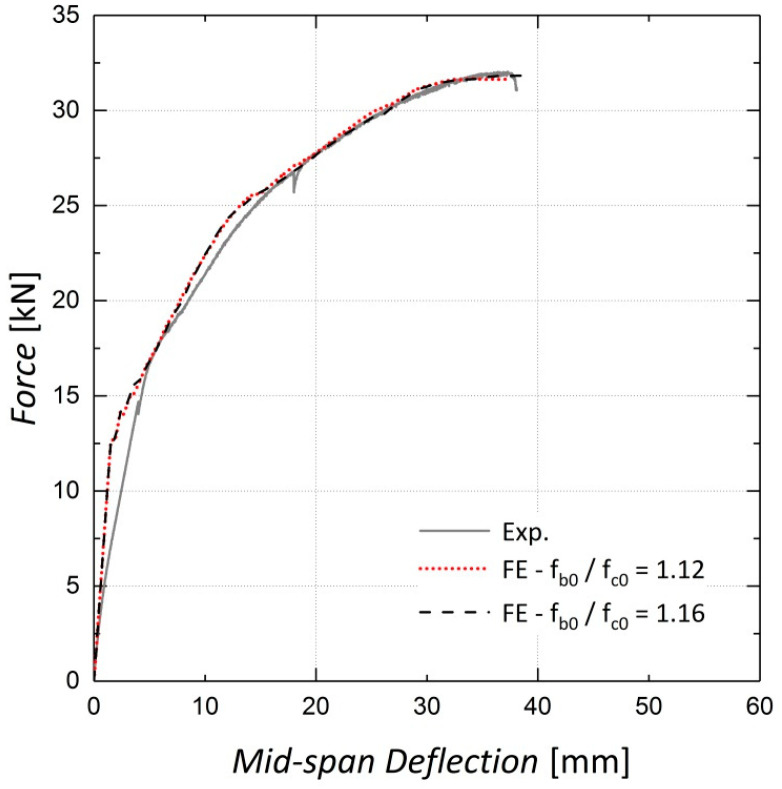
Sensitivity of results to f_b0_/f_c0_—Beam 11.

Sensitivity analyses were conducted to determine the appropriate values for some concrete damaged plasticity model parameters. The first investigated parameter was the ratio of the initial biaxial compressive strength to the uniaxial compressive strength, f_b0_/f_c0_, which was set to 1.16 according to the ABAQUS manual [22] as there were no biaxial compression test results available for the concrete used in the beams. As observed in Figure 6 and Figure 7, variations in this parameter have a negligible effect on the results because the elements are not primarily under biaxial compression during loading.

**Figure 8:**
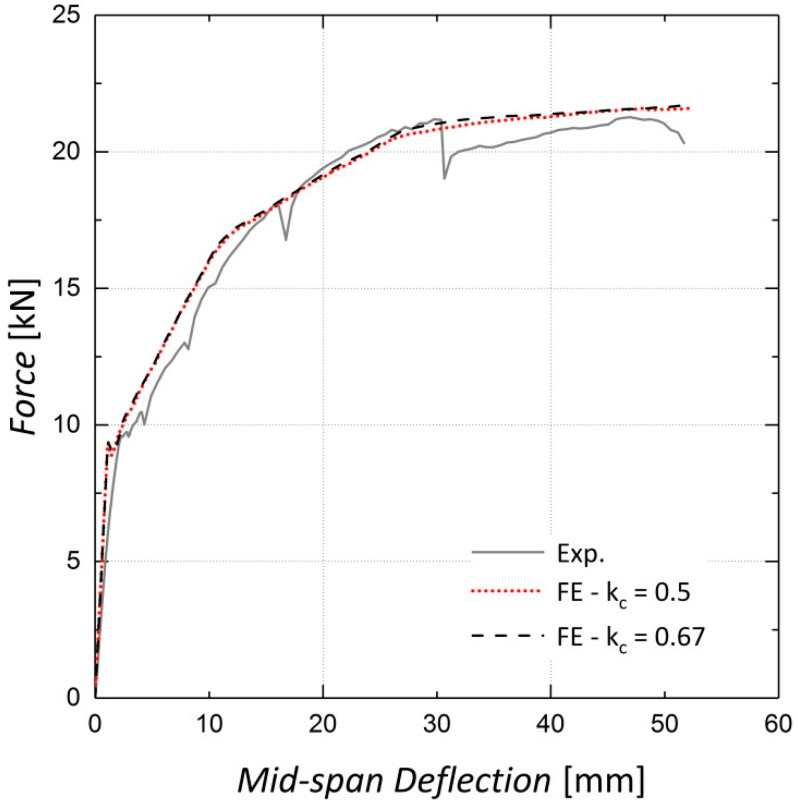
Sensitivity of results to k_c_—Beam 10.

**Figure 9:**
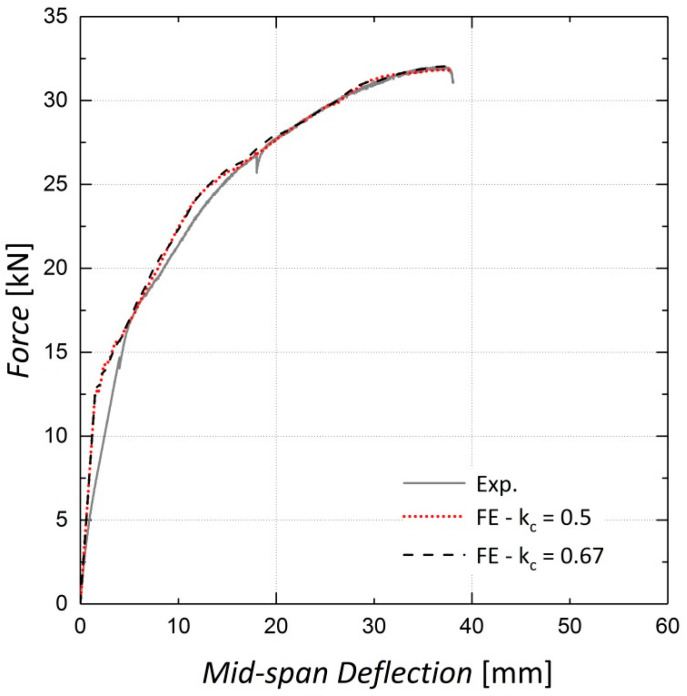
Sensitivity of results to k_c_—Beam 11.

**Figure 10:**
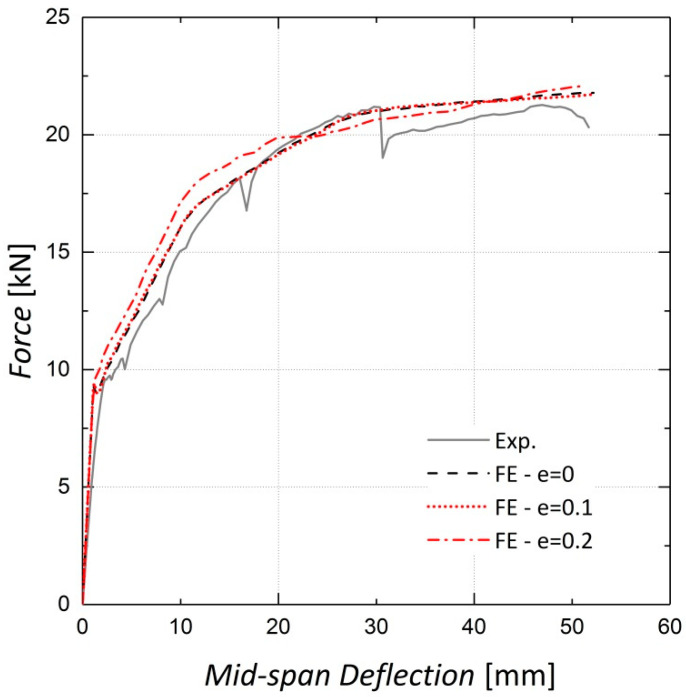
Sensitivity of results to eccentricity—Beam 10.

**Figure 11:**
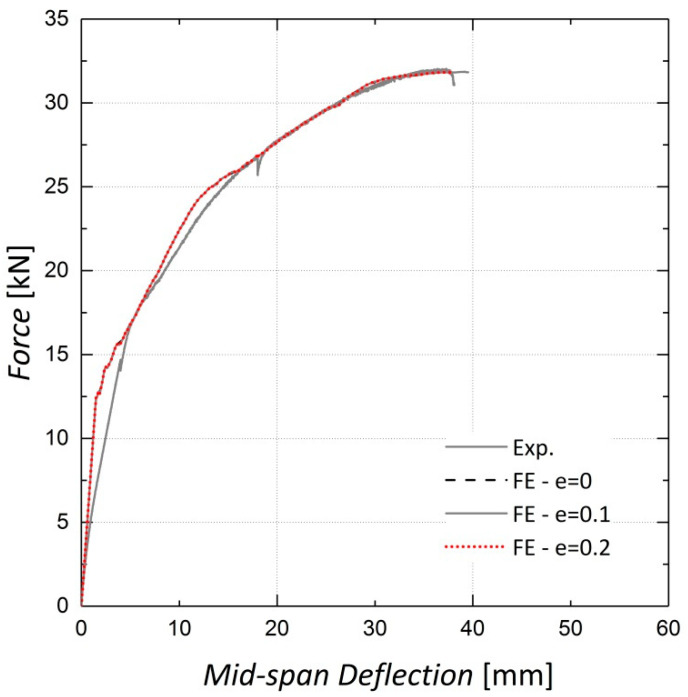
Sensitivity of results to eccentricity—Beam 11.

The shape of the loading surface k_c_ and the plastic potential eccentricity *e*, were set as 2/3 and 0.1, respectively, as suggested by the ABAQUS manual [22]. The k_c_ is determined from the results of the full triaxial test of concrete. However, these data were not available and the default values were assigned [22]. The results of the sensitivity analyses shown in Figure 8, Figure 9, Figure 10 and Figure 11 indicate no significant change in the load–displacement curves caused by variations in these two parameters; however, a small eccentricity value may lead to early convergence errors in the analysis.

**Figure 12:**
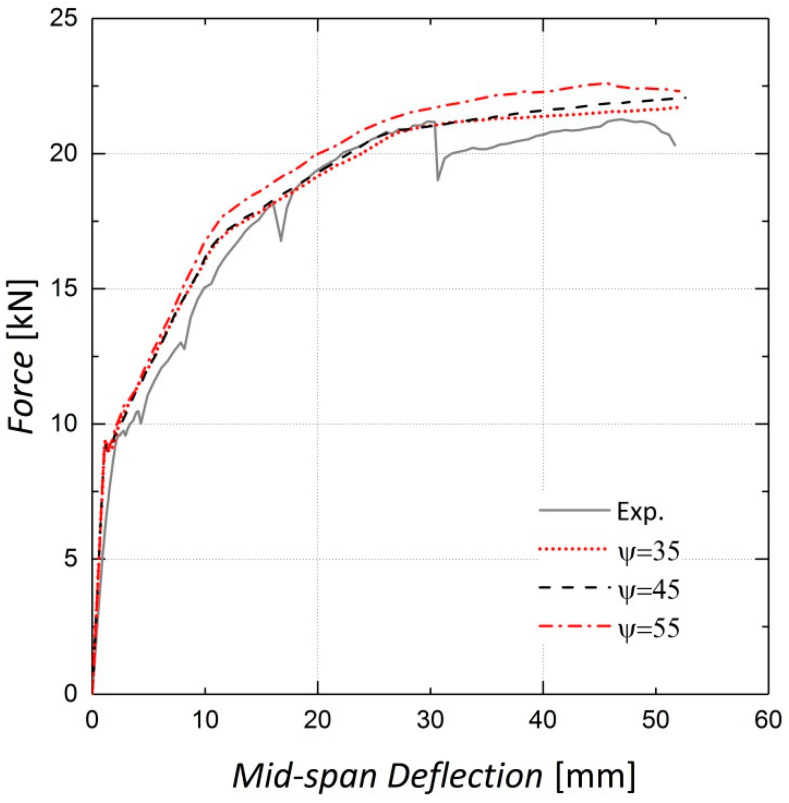
Sensitivity of results to dilation angle—Beam 10.

**Figure 13:**
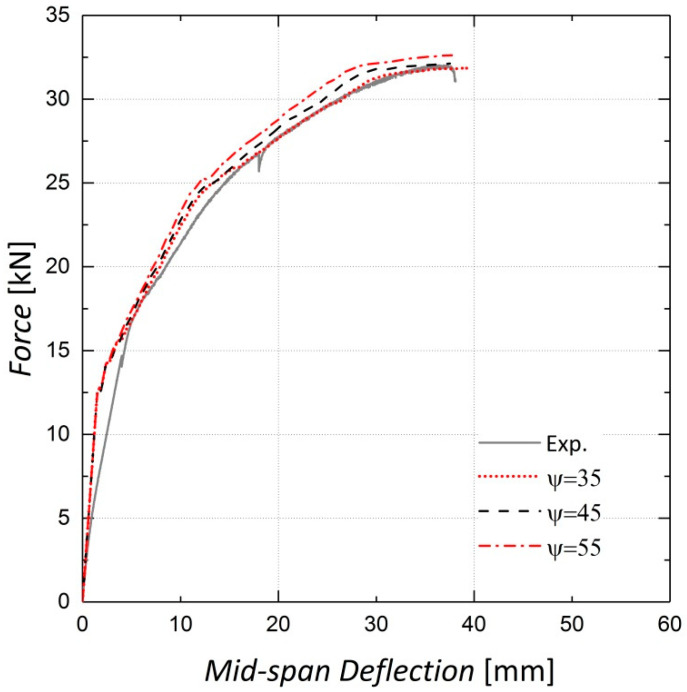
Sensitivity of results to dilation angle—Beam 11.

The concrete dilation angle ψ, which can be characterized as the internal friction angle of concrete, is an important parameter in the damaged plasticity model [23]. As shown in Figure 12 and Figure 13, adopting a greater dilation angle does not affect the load–displacement response of beams before cracking, although it increases the beam stiffness after cracking. According to [30], based on the analyses conducted for 35°, 45° and 55°, 35° was assigned to ψ for this analysis. 

**Figure 14:**
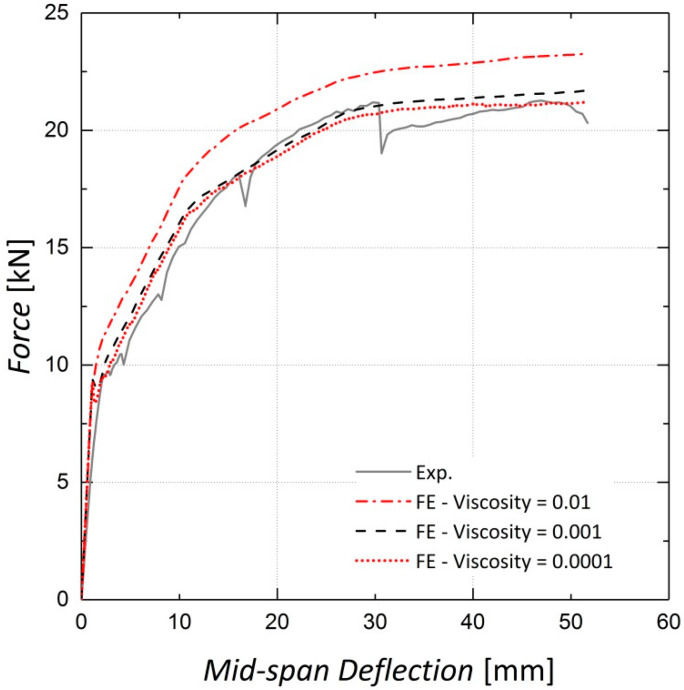
Sensitivity of results to viscosity—Beam 10.

**Figure 15:**
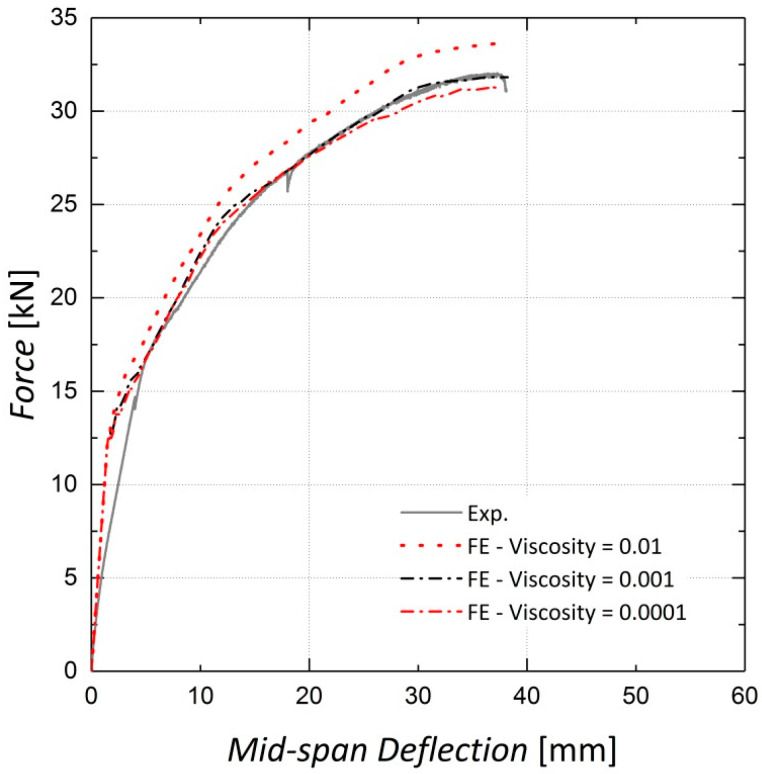
Sensitivity of results to viscosity—Beam 11.

Viscosity or relaxation time is an optional parameter in the CDP model and helps to improve the convergence rate in the analysis [22]. As stated by Szczecina et al. [31], this parameter seems to have a considerable effect on the results and should be chosen carefully. They proposed a value of 0.0001 for this parameter in their study [31]. The load–deflection results for Beams 10 and 11 for three different values of viscosity are presented in Figure 14 and Figure 15. It is observed that the results do not change considerably when the viscosity parameter is changed from 0.001 to 0.0001; however, as the value of 0.0001 leads to increased analysis time, that is, from two hours to four hours, the viscosity parameter is accordingly set to 0.001, as suggested by Yapar et al. [32]. 

#### 4.1.2. Load vs. Mid-Span Deflection Results 

**Figure 16:**
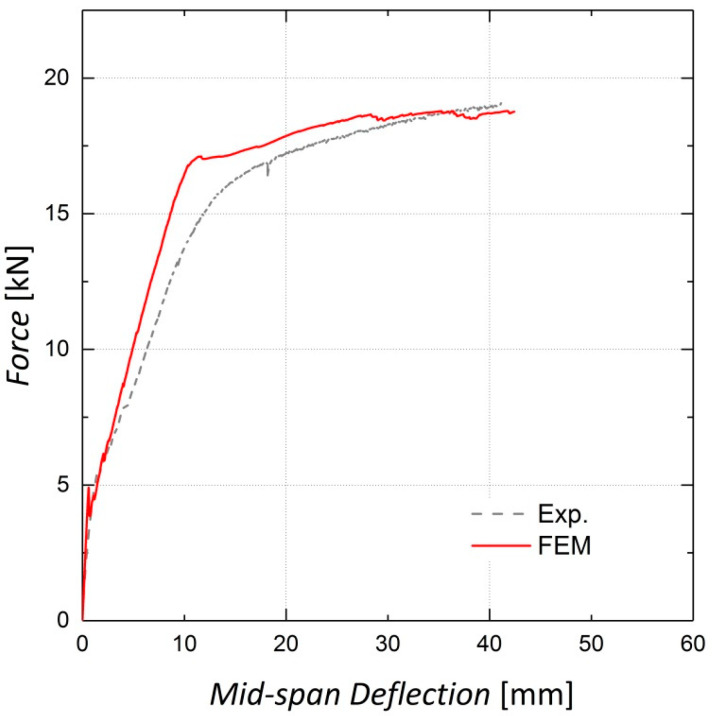
Finite Element analysis results for Beam 9 (non-prestressed).

**Figure 17:**
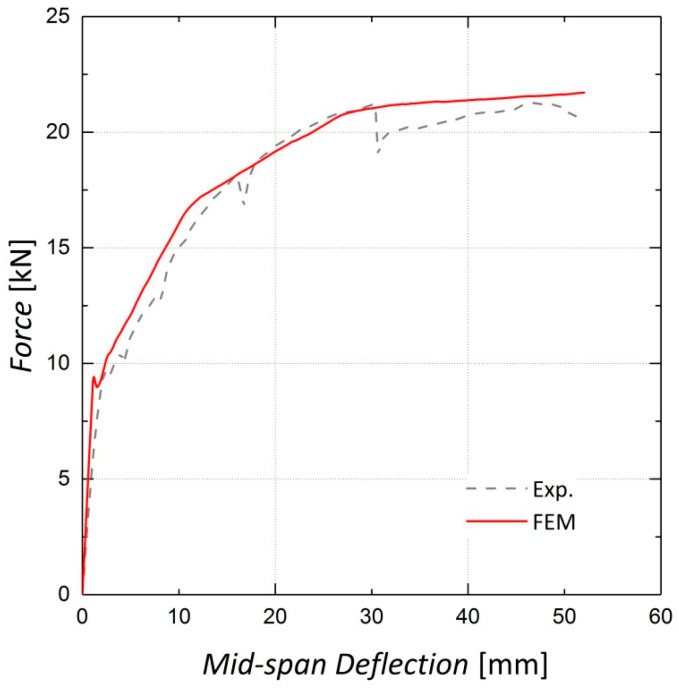
Finite Element analysis results for Beam 10 (pre-stressed by two Fe-SMA bars).

**Figure 18:**
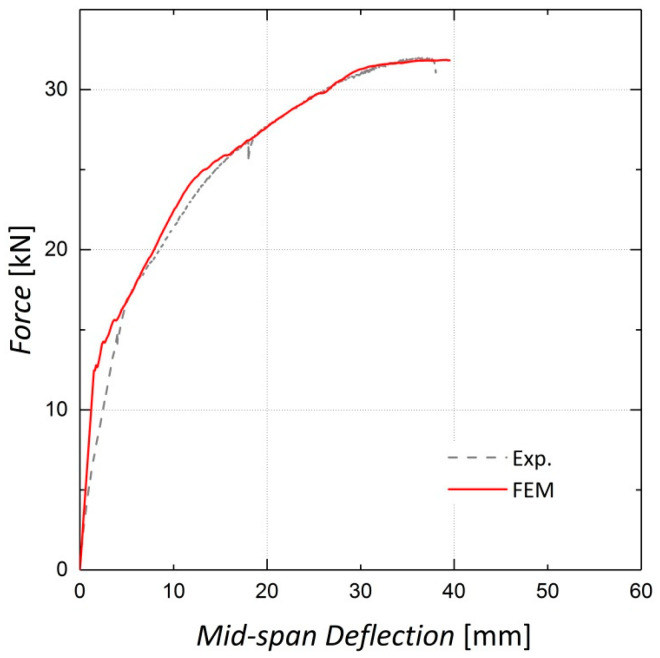
Finite Element analysis results for Beam 11 (pre-stressed by four Fe-SMA bars).

As the CDP parameters were chosen based on the sensitivity analysis, the finite element models were fully developed. Figure 16, Figure 17 and Figure 18 illustrate the finite element analysis results for Beams 9, 10 and 11, respectively, verified by the experimental data. These figures demonstrate that the damaged plasticity model combined with the predefined field option in ABAQUS can successfully model the behavior of pre-stressed RC beams. Despite the accuracy of the cracking load prediction in Beams 9 and 10, discrepancies are observed between the experimental and numerical values for the cracking load in Beam 11, which can be attributed to the presence of shrinkage cracks prior to loading [14]. For the ultimate load capacity of the beams, the results predicted by the finite element model exhibit an error of less than 1% for all three beams.

### 4.2. Parametric Studies

#### 4.2.1. Effect of Fe-SMA Diameter 

**Figure 19:**
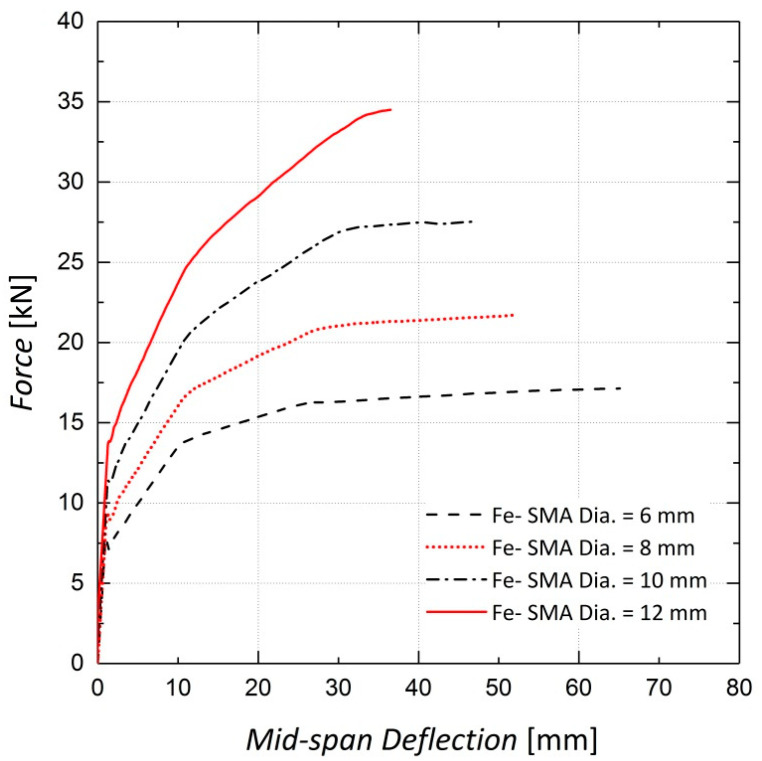
Sensitivity of results to Fe-SMA bar diameter—Beam 10.

**Figure 20:**
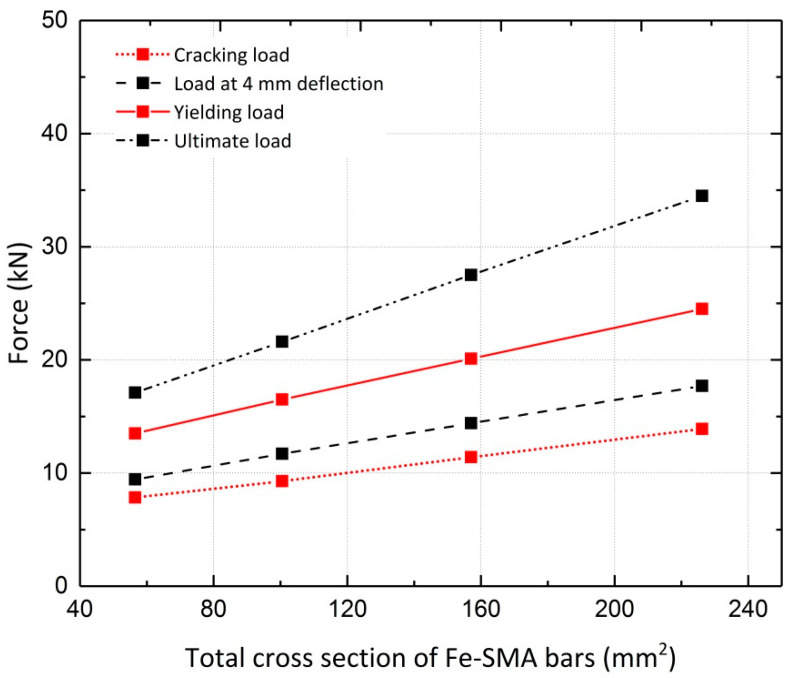
Comparison of results for four Fe-SMA bar diameters—Beam 10.

Pre-stressing concrete beams using Fe-SMA bars embedded in a shotcrete layer is state-of-the-art; the pros and cons have not been sufficiently studied. Thus, parametric studies are necessary to investigate the effect of variations in important parameters such as the diameter of the Fe-SMA bars on the response of beams, as shown in Figure 19. The cracking load, steel yielding load and ultimate load of the beams for different values of the Fe-SMA bar diameter are compared in Figure 20. The ultimate load exhibits the greatest increase with an increase in the Fe-SMA bar diameter. A simple calculation indicates that a four-fold increase in the cross-sectional area of the ribbed Fe-SMA bars, as a result of changing the Fe-SMA diameter from 6 mm to 12 mm, produces a 77%, 81% and 102% increase in the cracking load, steel yielding load and ultimate load of the pre-stressed beam, respectively. The loads corresponding to the serviceability limit state of 4 mm mid-span deflection for all Fe-SMA diameters considered in the analysis are also compared in Figure 20 [14].

The displacement ductility index $\mu_{\delta }$ describes the ability of the member to undergo additional deformations after being subjected to its maximum load capacity. The displacement ductility index is calculated by dividing the deflection at the ultimate load by the deflection at the steel yielding load, expressed as Equation (9). Table 1 shows the changes in the displacement ductility index of Beam 10 resulting from different Fe-SMA bar diameters. Increasing the bar diameter from 8 mm to 12 mm, representing a 125% increase in the cross-sectional area, decreases the displacement ductility index by 32%.

(9) $$\mu_{\delta }=\frac{\delta_{u}}{\delta_{y}}.$$

#### 4.2.2. Effect of Pre-Stressing Force

**Figure 21:**
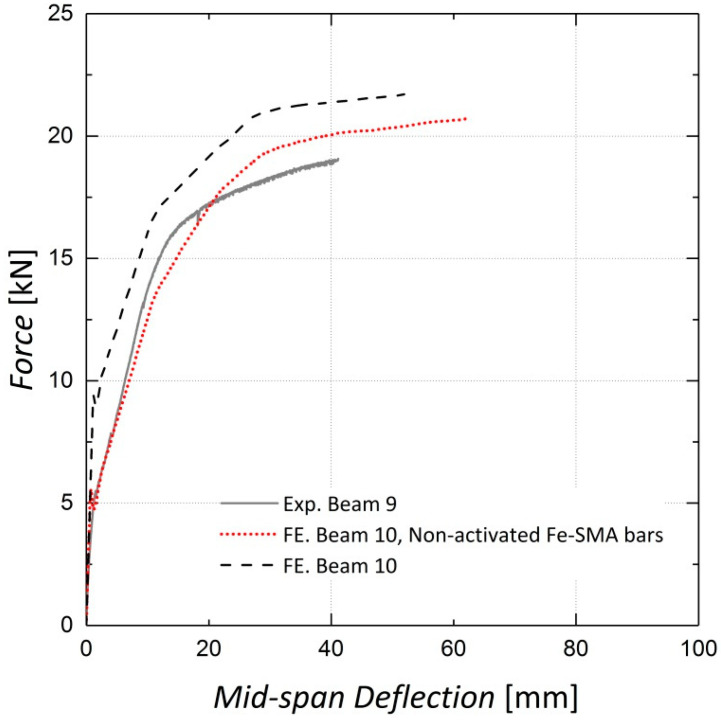
Effect of strengthening with activated and non-activated Fe-SMA bars—Beam 10.

Based on the innovative nature of this strengthening technique, the feasibility of using ribbed Fe-SMA bars to strengthen the beams without activating the recovery stress is investigated. The results are compared to those for the pre-stressed beam and the beam reinforced by two steel rebars embedded in the shotcrete layer, as shown in Figure 21 [13]. According to Figure 21, the activation of recovery stress in the strengthened beam with two Fe-SMA bars results in a 64.3% increase in the cracking load compared to the non-activated case; a 4.9% increase in the ultimate load was observed in the FE analysis results. The results also indicate that activating the recovery stress in the beam with two Fe-SMA bars leads to a 52.3% and a 50.7% increase in the yielding load and the serviceability limit state load, respectively. The calculated displacement ductility indices for beams strengthened with activated and non-activated Fe-SMA bars are presented in Table 2.

#### 4.2.3. Effect of Pre-Stressing Force Level

**Figure 22:**
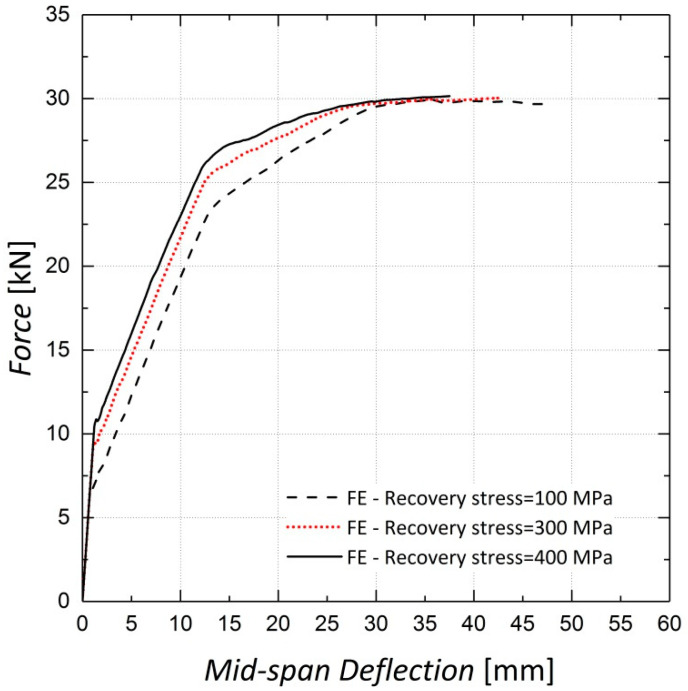
Load–deflection results—steel rebar diameter of 12 mm.

**Figure 23:**
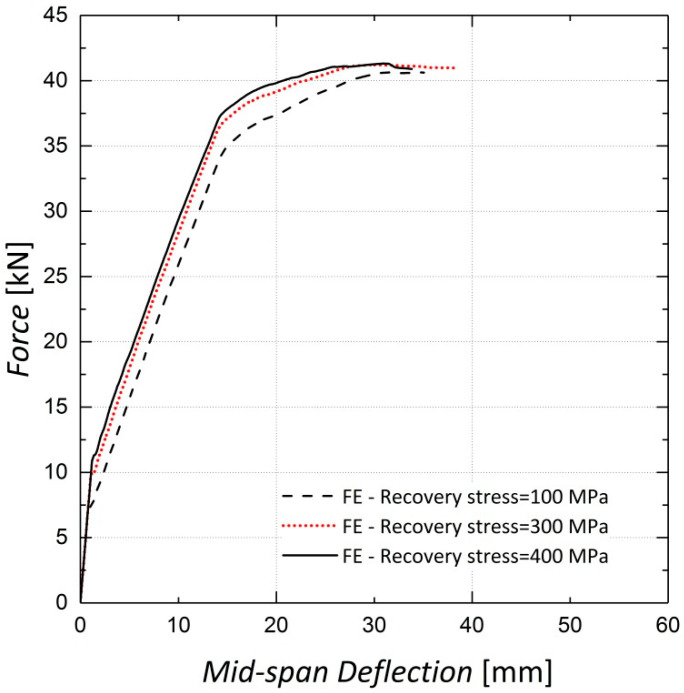
Load–deflection results—steel rebar diameter of 16 mm.

**Figure 24:**
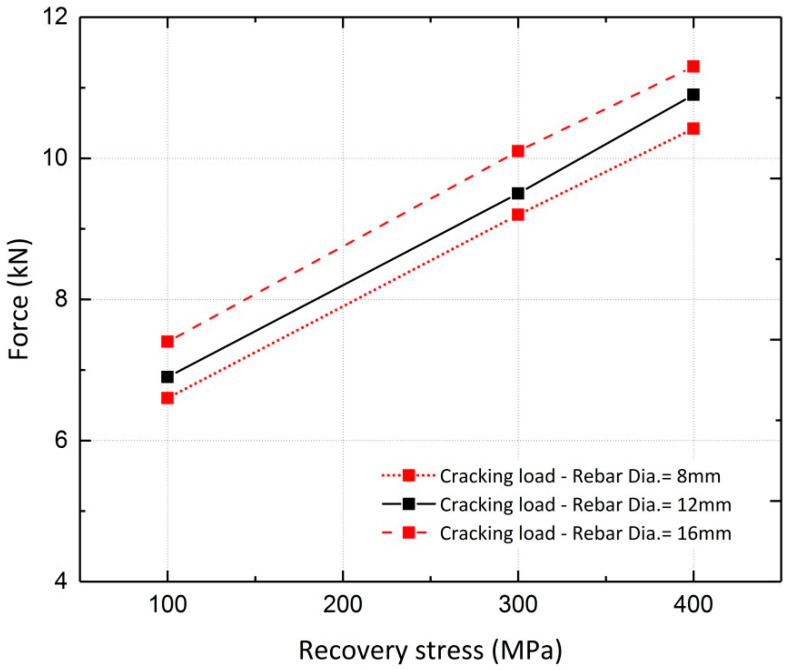
Comparison of cracking loads.

**Figure 25:**
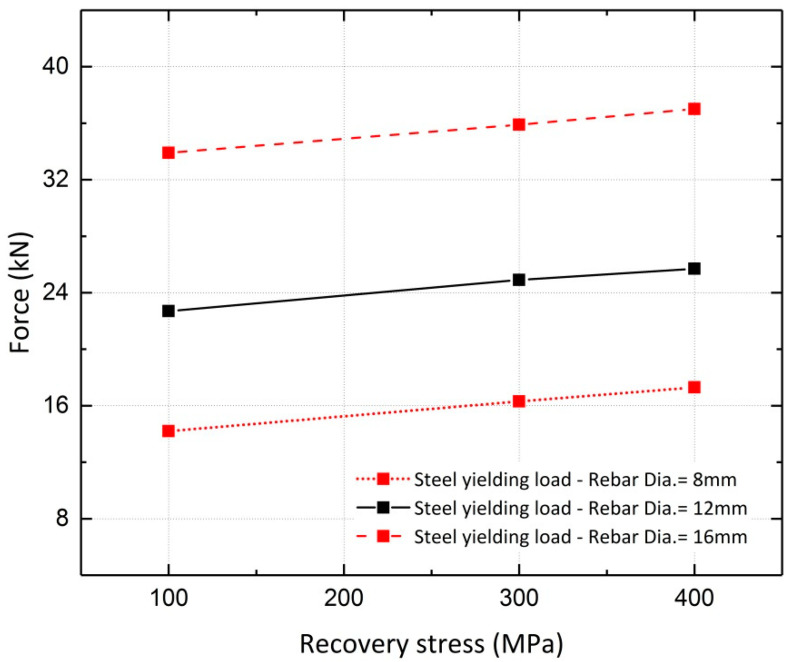
Comparison of steel yielding loads.

**Figure 26:**
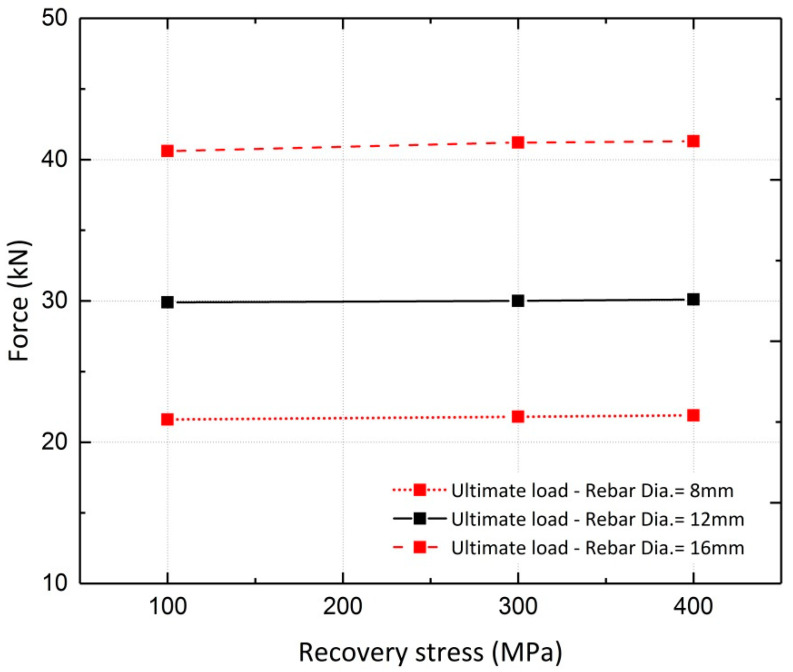
Comparison of ultimate loads.

A parametric study was conducted to investigate the effect of the recovery stress value on the beam flexural response. This study was conducted on the FE model of Beam 10, with different diameters assigned to the longitudinal steel reinforcement. Beams with reinforcement ratios (*ρ)* of 0.34%, 0.75% and 1.3%, corresponding to rebar diameters of 8 mm, 12 mm and 16 mm, were analyzed. The analyses were conducted for different levels of pre-stressing, 100 MPa, 300 MPa and 400 MPa. Figure 22 and Figure 23 illustrate the results for beams reinforced with rebar diameters of 12 mm and 16 mm, respectively. It can be inferred from the results that the variation in recovery stress has a more significant effect on the beam with a reinforcement ratio of 0.75%. The cracking load, steel yielding load and ultimate load values were extracted from the curves and are shown in Figure 24, Figure 25 and Figure 26, respectively. The values extracted from the curves demonstrate that increasing the pre-stressing force leads to a noticeable increase in the cracking loads; however, the ultimate loads do not change significantly, which demonstrates the point in Figure 21, that is, activation of Fe-SMA recovery stress does not have a considerable impact on the value of the ultimate load of the beams. Changing the recovery stress from 100 MPa to 400 MPa in the beam with a reinforcement ratio of 0.34% leads to a 57.6%, 21.8% and 1.4% increase in the cracking load, yielding load and ultimate load, respectively. In other words, the application of conventional pre-stressing (using tendons, etc.) mainly affects the serviceability state while it does not seem to have a considerable impact on the ultimate limit state.

Based on the results illustrated in the previous section, variations in the load–deflection results due to changes in the longitudinal rebar diameter can also be investigated. Increasing the reinforcement ratio has a more significant effect on the steel yielding load than on the ultimate and cracking loads. With a pre-stressing force of 300 MPa, the cracking, steel yielding and ultimate loads of the beams increase by approximately 9.8%, 120.2% and 89.0%, respectively, when the reinforcement ratio varies from 0.34% to 1.3%. For this value of recovery stress, the calculated displacement ductility indices for the three reinforcement ratios (0.34%, 0.75% and 1.3%) are 5, 3.5 and 2.2, respectively.

### 4.3. Case Study, Numerical Modeling of Bridge Girder Pre-Stressed by Fe-SMA Bars in a Shotcrete Layer

**Figure 27:**
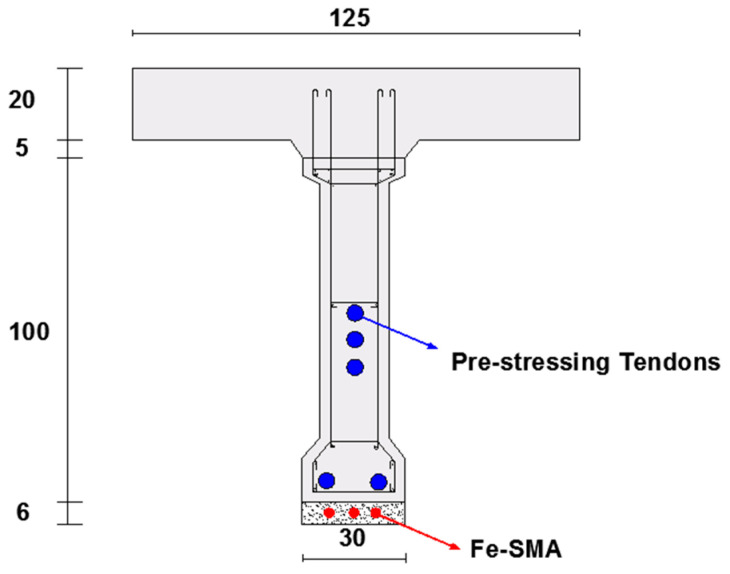
Longitudinal profile and cross-section of the girders (in cm) [33].

In previous sections, the feasibility of strengthening and pre-stressing reinforced concrete beams using ribbed Fe-SMA bars embedded in a shotcrete layer was studied by means of numerical analysis. The validated 3-D finite element models are used in this section in a case study on the load-bearing capacity of an 18m reinforced and pre-stressed concrete bridge girder strengthened by activated or non-activated Fe-SMA bars embedded in a shotcrete layer. The reference girder, used for comparison, is characterized by a double-tee section with a height of 1 m and is pre-stressed by three parabolic and two straight steel tendons, each with a 345 mm^2^ cross-sectional area. A 1.25 m × 0.2 m upper concrete slab was also modeled. The cross-section schematic and longitudinal profile of the reference girder are illustrated in Figure 27 [33].

The depth of the assumed bottom shotcrete layer was chosen as 40 mm. The shotcrete had the same material properties as the shotcrete applied to the beams in the previous sections. Key parameters defining the mechanical properties of concrete, longitudinal steel bars and tendons are presented in Table 3 [33]. Five different girders strengthened by different arrangements of Fe-SMA bars embedded in the bottom shotcrete layer are studied in this section. Table 4 presents the calculated total cross-sectional area of the Fe-SMA bars used with each girder.

**Figure 28:**
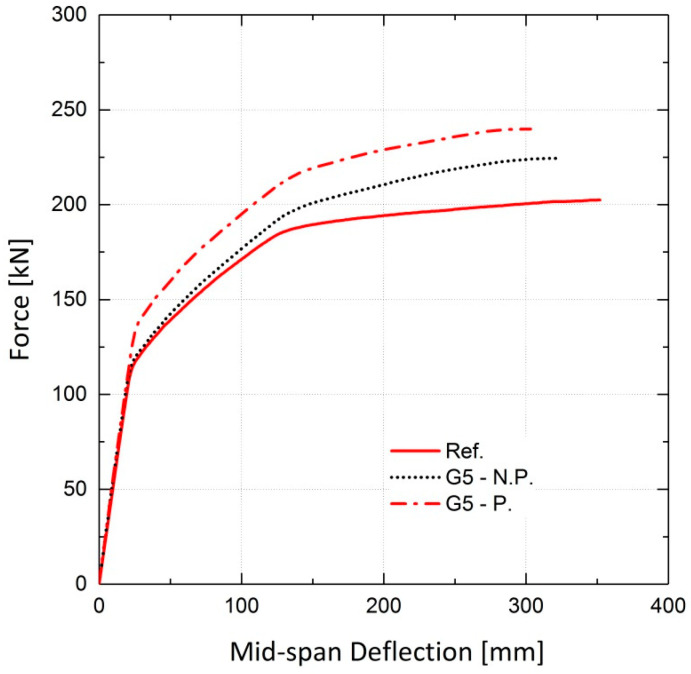
Effect of activated and non-activated Fe-SMA bars on load–deflection behavior of G5.

**Figure 29:**
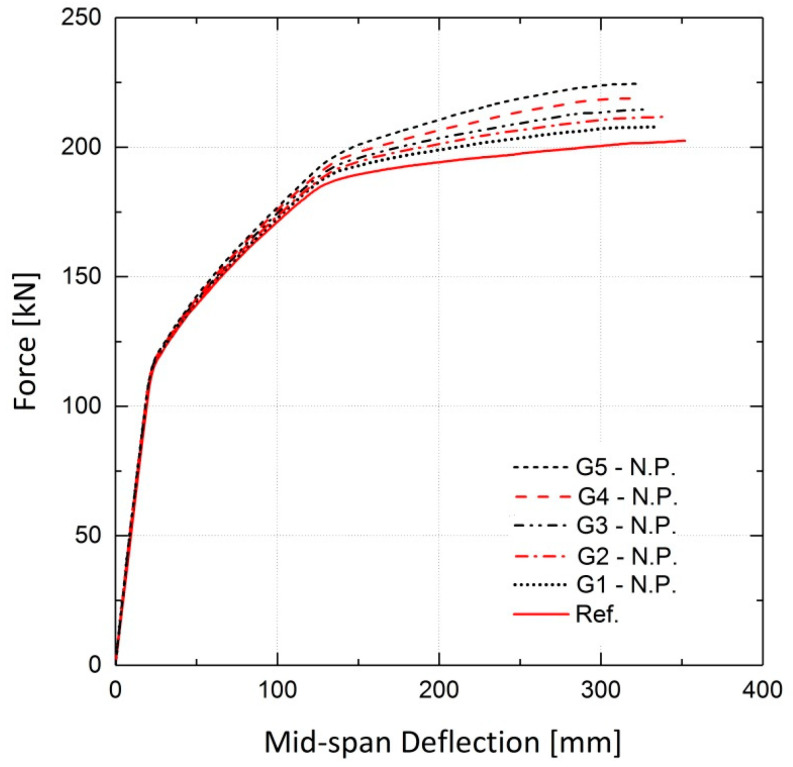
Effect of non-activated Fe-SMA bars on load–deflection behavior of girders compared to the reference.

**Figure 30:**
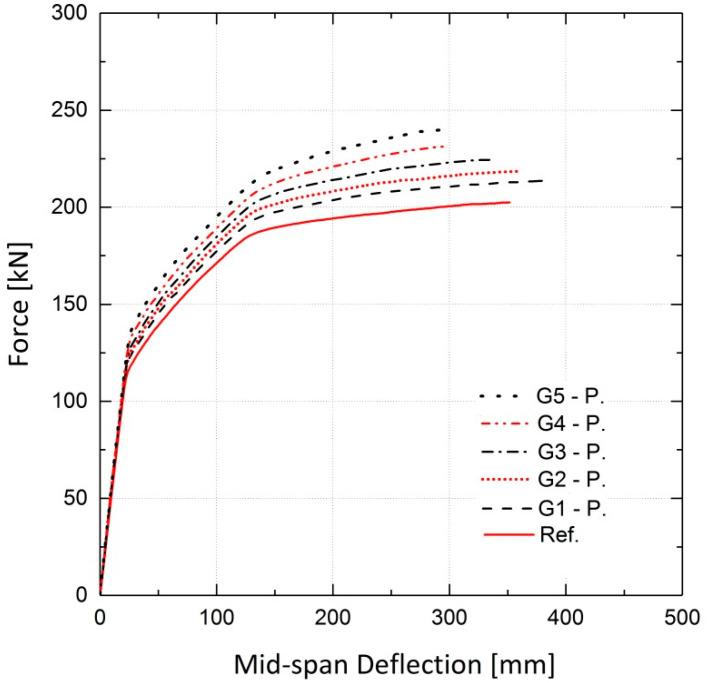
Effect of activated Fe-SMA bars on load–deflection behavior of girders compared to the reference.

**Figure 31:**
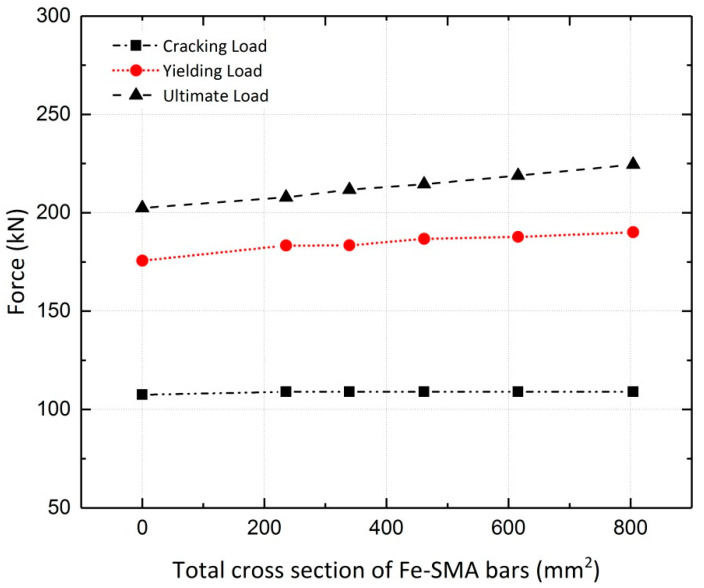
Effect of non-activated Fe-SMA bars on cracking, yielding and ultimate loads of girders.

**Figure 32:**
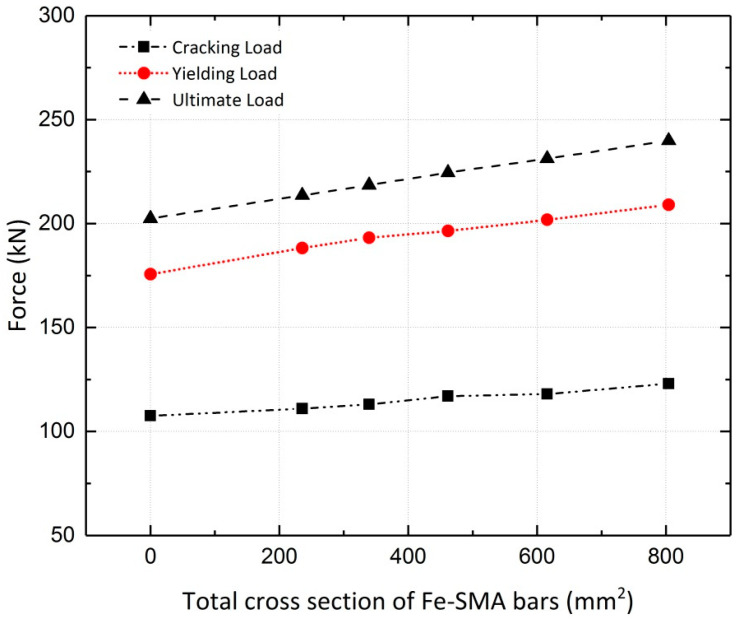
Effect of activated Fe-SMA bars on cracking, yielding and ultimate loads of girders.

The simulated girders, strengthened by activated or non-activated Fe-SMA bars, were analyzed under static loading conditions. The results are compared to those for the non-strengthened girder (reference girder) in terms of the load vs. mid-span deflection curves. The results of the finite element analysis for girder G5 are illustrated in Figure 28, demonstrating the effect of this strengthening method on the load-bearing capacity of the girders. It can be inferred that application of activated Fe-SMA bars led to a 19% and 18.5% increase in the yielding and ultimate loads of G5, respectively, compared to 8.2% and 10.9% increases in the non-prestressed G5. Similar to the results of the beam analysis in Section 4.2.2, it is concluded that the application of non-prestressed Fe-SMA bars has almost no effect on the cracking load of the girders. It can also be further deduced that using activated or non-activated Fe-SMA bars does not make a major difference in the value of the ultimate load of girders. For the rest of the girders in Table 4, the results for non-prestressed and pre-stressed girders are illustrated in Figure 29 and Figure 30, respectively. The results for the cracking load, yielding load and ultimate load of the girders are shown in Figure 31 and Figure 32. 

It can be concluded that application of activated Fe-SMA bars has a more considerable impact on cracking and yielding load of girders compared to the girders strengthened with non-activated bars. This advantage of activation must be evaluated considering the equipment and cost of activating bars for such massive structures. 

To recap, the case study in this paper aimed to investigate the feasibility of strengthening concrete bridge girders with Fe-based alloys. The finite element simulation results were promising; however, the applicability of the proposed strengthening method should be tested on several girder geometries with different reinforcement levels. Also, the proposed method’s applicability under different loading patterns could be an essential subject of further studies.

## 5. Conclusions

In this study, numerical analyses of concrete beams strengthened and pre-stressed with Fe-based shape memory alloy bars were conducted. The constitutive models used for each material in the RC beam were explained along with the proper approach to implementing the pre-stressing effect on the beams in the 3-D finite element model. The results of the analyses compared to the experimental data indicate that the finite element models successfully predicted the load vs. mid-span displacement response of the beams. In addition, sensitivity analyses were performed to study the effect of variations in parameters in the concrete damaged plasticity model in ABAQUS. The results indicate that changes in eccentricity, k_c_ and the ratio of the biaxial compressive strength to the uniaxial compressive strength did not significantly affect the load–displacement response of the models; different concrete dilation angles yielded different results in the post-cracking stage. Adopting a proper viscosity parameter had a positive effect on the convergence and accuracy of the analysis results.

Finite element analyses conducted for beams reinforced with different ratios of longitudinal rebars pre-stressed with three values of recovery stress activated in the Fe-SMA bars indicate that variation in the pre-stressing force produces a greater effect on a beam with a lower reinforcement ratio *(ρ* varies from 0.34–1.3%). Moreover, the variation in the recovery stress affects the cracking load more significantly than the yielding load and the ultimate load.

The flexural behavior of a similar beam strengthened by non-activated Fe-SMA bars was also investigated and compared to the pre-stressed and reference beams. The results indicate that activating the recovery stress increases the ultimate load by less than 5% compared to the non-prestressed beam; however, the cracking and yielding loads increase significantly. 

The feasibility of pre-stressing an 18-m concrete bridge girder by means of activated and non-activated Fe-SMA bars embedded in a shotcrete layer was studied. It was concluded that the load-bearing capacity of the bridge girders was significantly enhanced when using this method with Fe-SMA bars of a sufficient number and diameter.

## Figures and Tables

**Table 1:** Values of $\mu_{\delta }$ for Beam 10 pre-stressed by different diameters of Fe-SMA bar.

Fe-SMA Bar Diameter	Displacement Ductility Index
6 mm	6.6
8 mm	4.9
10 mm	4.4
12 mm	3.6

**Table 2:** Comparison of beams strengthened with activated and non-activated Fe-SMA bars.

Parameter	Non-Activated Fe-SMA Bars	Activated Fe-SMA Bars
Cracking load (kN)	5.6	9.2
Load at 4 mm mid-span deflection (kN)	7.5	11.3
Bottom steel rebar yielding load (kN)	10.7	16.3
Ultimate load (kN)	20.7	21.8
Displacement ductility Index	7.9	5

**Table 3:** Mechanical properties of concrete, longitudinal steel rebar and tendons [33].

Parameter	Value
Cubic compressive strength of concrete—girder (MPa)	64.6
Cubic compressive strength of concrete—slab (MPa)	50.0
Elastic modulus of concrete—girder (GPa)	34.7
Elastic modulus of concrete—slab (GPa)	32.1
Elastic modulus of steel rebar (GPa)	200
Elastic modulus of steel tendon (GPa)	210.3
Yield stress of steel rebar (MPa)	462
Yield stress of steel tendon (MPa)	1660
Tensile strength of steel rebar (MPa)	545
Tensile strength of steel tendon (MPa)	1810
Strain at failure of steel rebar (%)	10.6
Strain at failure of steel tendon (%)	3.76

**Table 4:** Cross-sectional area of different configurations of Fe-SMA bars in girders.

Girder Name	Total Cross-Sectional Area of Fe-SMA Bars (mm^2^)
Reference	none
G1	235.6
G2	339.3
G3	461.8
G4	615.8
G5	804.2
